# Antithrombotic therapy with rivaroxaban in five patients with paroxysmal nocturnal haemoglobinuria and thrombotic events

**DOI:** 10.1186/s12959-018-0181-5

**Published:** 2018-10-22

**Authors:** Francesco Dragoni, Flavia Chiarotti, Anna Paola Iori, Ursula La Rocca, Arturo Cafolla

**Affiliations:** 1 Thrombosis Center, “Department of Cellular Biotechnologies and Hematology”, “Sapienza” University, Via Benevento 6, 00161 Rome, Italy; 2 Reference Center for Behavioral Sciences and Mental Health, National Institute of Health, Rome, Italy

**Keywords:** Paroxysmal nocturnal haemoglobinuria, Antithrombotic therapy, Rivaroxaban, Vitamin K antagonist, Thrombotic events

## Abstract

Five patients with paroxysmal nocturnal haemoglobinuria and thrombotic complications under oral antithrombotic treatment with vitamin K antagonist were switched to receive the direct oral anticoagulant rivaroxaban an factor Xa inhibitor. In all five patients haematological and biochemical parameters and adverse events were evaluated for a period of twelve months.

Therapy with rivaroxaban was well tolerated in all cases and one patient showed a significant reduction of bleeding and transfusion requirement. All patients obtained a significant reduction in days of hospitalization with a consequent improvement in their quality of life after rivaroxaban treatment.

Paroxysmal nocturnal haemoglobinuria (PNH) is a rare acquired clonal disorder of hematopoietic cells characterized by a defect in the glycosylphosphatidylinositol (GPI) anchored molecules due to a somatic mutation in the X linked PIG-A gene. This leads to a partial or complete absence of two complement inhibitor GPI-linked proteins, particularly CD59 and CD55, from the membrane of the circulating cells, causing the activation of the complement system on red cells surface and intravascular haemolysis [1].

In addition to intravascular haemolytic anaemia, PNH is characterized by hypercoagulable state and bone marrow aplasia. The hypercoagulable state is responsible for life-threatening venous thrombosis generally arising in hepatic, intra-abdominal, cerebral and extremity veins and these thrombotic complications represent the most common cause of death in patients with PNH [2].

Prophylactic anticoagulant treatment seems capable of improving survival and morbidity [3]. Moreover, eculizumab seems able to prevent thrombosis as an indirect effect on intravascular haemolysis [4] and to reduce the activation of coagulation [5].

We report the outcome of haematological and biochemical parameters, recurrence of thrombotic and bleeding events in five patients (Males = 1, Females = 4, mean age 47.4 years) with PNH and thrombotic complications evaluated for a period of twelve months both during warfarin, a vitamin K antagonist (VKA), and rivaroxaban treatment. Moreover, the assessment of occult blood in the stool was performed monthly in all five evaluated patients. These five patients were being treated with the monoclonal antibody eculizumab administered every 15 days during the both VKA and rivaroxaban treatment.

Two patients received rivaroxaban at dosage of 15 mg per day for previous bleeding complications observed during the treatment with warfarin. The remaining three patients received rivaroxaban at a dosage of 20 mg per day.

The quantitative determination of rivaroxaban concentrations was assessed after 3 months of therapy using an anti-factor Xa chromogenic assay (Biophen DiXal, Hyphen Biomed, France). Patients who received rivaroxaban 15 mg per day showed, after 20 h from the drug administration, a concentration of 10 and 14 ng/mL, respectively. The remaining three patients who received 20 mg per day of rivaroxaban treatment had, after 20 h from the drug administration, concentrations of 17, 27 and 41 ng/mL, respectively. Therefore two patients received a reduced dosage of rivaroxaban which, however, can be utilized at this lower dose in patients with high risk of haemorrhagic complications or impaired renal function.

Since thrombotic complications occur frequently in unusual sites in patients with PNH, as reported in Table 1, and rivaroxaban has no indication to be used in cerebral, caval or portal vein thrombosis, we used, in this study, the new oral direct anticoagulant in off label indications.

**Table 1:** Outcome of the evaluated parameters during twelve months of anticoagulant treatment with warfarin and rivaroxaban in the patients with paroxysmal nocturnal haemoglobinuria

	W	R		W	R		W	R	
	Pt 1: Female, 45 yrs.			Pt 2: Female, 66 yrs.			Pt 3: Female, 45 yrs.		
	Thrombosis of superior sagittal sinus			Thrombosis of portal vein			Deep vein thrombosis		
Haemoglobin (gr/dL)	7. 7± 0.4	8.4 ± 0.6	0.006	12.5 ± 0.6	13.1 ± 0.2	n. s.	7.9 ± 0.5	7.8 ± 0.6	n. s.
Haematocrit (%)	22.9 ± 2	25.4 ± 1.4	0.003	38.5 ± 1.4	39.4 ± 0.7	n. s.	27.8 ± 1.6	28.6 ± 2.8	n. s.
Platelets (× 10^3^/μL)	81.9 ± 9.4	73.8 ± 10.3	n. s.	118.1 ± 24.2	112.3 ± 6.7	n. s.	357.2 ± 27.2	347.3 ± 28.8	n. s.
Lactate deydrogenase (IU/L)	326.7 ± 15.0	311.7 ± 11.4	n. s.	249.1 ± 44.4	257.3 ± 40.1	n. s.	331.6 ± 207.3	312.8 ± 177.3	n. s.
Bleeding Days	6.0 ± 0.8	3.6 ± 0.7	0.001						
Low Molecular Weight Heparin (Days of bridging-therapy)	8.2 ± 0.7	1.7 ± 1.1	< 0.001						
Red Blood Cell Units transfused	11	4	0.03	0	0		6	6	
Diapers (number)	14.9 ± 0.6	10 ± 0.8	< 0.001						
Hospital admissions (Days per month)	4.2 ± 0.7	2.7 ± 0.8	< 0.001	3.7 ± 0.6	2.8 ± 0.6	0.001	4.0 ± 0.7	2.6 ± 0.8	0.001
	Pt 4: Male, 27 yrs.			Pt 5: Female, 54 yrs.			All patients		
	Thrombosis of portal vein			Thrombosis of caval vein					
Haemoglobin (gr/dL)	13.3 ± 0.6	13.6 ± 0.9	n. s.	10.9 ± 0.3	10.5 ± 0.2	n. s.	9.9 ± 2.5	9.8 ± 2.4	n. s.
Haematocrit (%)	39.9 ± 1.9	39.3 ± 3.3	n. s.	33.7 ± 1.4	31.0 ± 1.0	n. s.	31.4 ± 7.0	30.5 ± 5.7	n. s.
Platelets (× 10^3^/μL)	94.3 ± 9.9	94.4 ± 11.5	n. s.	186.7 ± 23.9	192.5 ± 13.9	n. s.	186.3 ± 120.5	191.9 ± 123.0	n. s.
Lactate deydrogenase (IU/L)	228 ± 28.1	200.7 ± 21.4	n. s.	310.3 ± 57.4	272.0 ± 6.0	n. s.	285.9 ± 107.3	274.4 ±100.0	n. s.
Bleeding Days									
Low Molecular Weight Heparin (Days of bridging-therapy)									
Red Blood Cell Units transfused	0	0		0	0		17	10	n. s.
Diapers (number)									
Hospital admissions (Days per month)	3.5 ± 0.5	2.2 ± 0.4	0.001	3.1 ± 0.5	2.2 ± 0.6	< 0.001	3.7 ± 0.7	2.5 ± 0.7	< 0.001

Reported data are the mean values and standard deviations of the evaluated parameters performed every month for twelve months during the treatment with warfarin (W) and rivaroxaban (R)

Statistical analysis was performed using Student’s t-test. Significant level was set to *p* < 0.05. n. s. = not significant

Haemoglobin levels and haematocrit values were stable during the 12 months of rivaroxaban treatment (Table 1). None out of five patients with PNH discontinued the therapy with rivaroxaban for the occurrence of both thrombotic or bleeding events. Patients one (Table 1) is a young female patient with PNH and thrombotic complication who after the beginning of anticoagulant treatment with warfarin showed major bleeding due to severe menorrhagia that required red blood cell concentrates transfusion even if the PT INR value was in the established range. In this patient the administration of rivaroxaban instead of warfarin led to a significant reduction in haemorrhagic complication and transfusion requirement with a significant improvement in her quality of life.

It is important to consider that patients with PNH are forced to have a high number in days of hospitalization per month in order to monitor the haematological parameters, to receive eculizumab therapy and to check the anticoagulant treatment with VKA. The five patients on therapy with rivaroxaban showed a significant reduction in days of hospitalization per months due to reduced controls for follow-up of the new antithrombotic treatment (Table 1).

The quality of life (QOL) total score and the QOL scores for each domain was determined in four out of our five patients according to WHOQOL-BREF questionnaire [6] since one patient refused the interview (Table 2). As for as the quality of life is concerned, the total WHOQOL score was higher after rivaroxaban therapy than before it, similarly scores for the physical (7 items), psychological (6 items), social relationship (3 items) and environment (8 items) domains. As concerns the physical and psychological domains the increase in QOL due to rivaroxaban was statistically significant. After twelve months of therapy with rivaroxaban all patients with PNH refused to resume the previous treatment with VKA.

**Table 2:** The World Health Organization Quality of Life (WHOQOL-BREF) after 12 months of anticoagulant treatment with warfarin and rivaroxaban in the patients with paroxysmal nocturnal haemoglobinuria

	Pt	1	Pt	2	Pt	3	Pt	4	All patients (*n* = 4)		t-test
	W	R	W	R	W	R	W	R	W	R	p
Total (26 items)	67	76	42	65	47	58	62	67	54.5 ± 11.9	66.5 ± 7.4	0.053
QOL rate (1 item)	25	75	50	50	25	25	25	75	31.3 ± 12.5	56.3 ± 23.9	n. s.
Satisfaction (1 item)	50	75	50	25	25	25	50	50	43.8 ± 12.5	43.8 ± 23.9	n. s.
Physical (7 items)	64	71	29	54	32	50	46	61	42.8 ± 16.0	59.0 ± 9.2	0.022
Psychological (6 items)	71	83	50	67	46	58	63	67	57.5 ± 11.6	68.8 ± 10.4	0.025
Social relationships (3 items)	83	83	50	67	92	92	67	75	73.0 ± 18.5	79.3 ± 10.7	n. s.
Environment (8 items)	69	72	44	81	50	59	78	72	60.3 ± 15.9	71.0 ± 9.1	n. s.

*W* Warfarin treatment; *R* Rivaroxaban treatment. Statistical analysis was performed using Student’s t-test

Significant level was set to *p* < 0.05. n. s. = not significant

The anticoagulant therapy in patients with PNH is particularly complex since these patients can develop the aplastic anemia with consequent thrombocytopenia which increases bleeding risk above all in patients under antithrombotic treatment. In addition, the multi-drugs therapy in patients with PNH interferes with the management of antithrombotic therapy, although the thrombosis prophylaxis and treatment are mandatory for these patients.

Until now, the experience on the use of direct oral anticoagulant drugs in patients affected by PNH is low and therefore, we suggested to discuss the use of these new drugs in these specific patients.

Rivaroxaban is an orally active direct Factor Xa inhibitor, which demonstrated to be effective in the prevention of venous thromboembolism after orthopedic surgery [7]. For people with non-valvular atrial fibrillation rivaroxaban was non-inferior to warfarin to prevent stroke or embolism, with significant reduction in intracranial haemorrhage and fatal bleeding [8]. Rivaroxaban can be given in fixed doses and without routine anticoagulant monitoring offering a less complex therapeutic regimen for patient. The new direct oral anticoagulant drugs, anti FIIa and FXa (Dabigatran, rivaroxaban, apixaban, and edoxaban) are effective in the prophylaxis and treatment of deep vein thrombosis and pulmonary embolism with a low incidence of severe haemorrhagic complications respect to VKA. In addition, recently low dose of rivaroxaban instead of the classical dosage was effective in reducing recurrent venous thromboembolism among patient who were in equipoise for continued anticoagulation without to modify bleeding risk [9]. In agreement with these data, it is interesting to note that the two patients with PNH who received a lower dose of rivaroxaban (15 mg per day) did not have thrombotic complications and did not interrupt the therapy for the occurrence of adverse events during twelve months of treatment.

These results suggest that rivaroxaban at the standard dose or at the low dose could have a role in selected patients with a high risk to develop bleeding complications during the treatment with antithrombotic drugs such as patients affected by PNH.

The improvement in the quality of life observed in our patients with PNH, who know very well the characteristics of their disease, seems to be due to the awareness that rivaroxaban therapy reduces significantly both severe bleeding complications and days of hospitalization.

In conclusion this report suggests that rivaroxaban therapy appears to be effective and safe in the treatment of patients with PNH. Moreover, rivaroxaban was able to ameliorate the quality of life in these specific patients. However, further studies are needed to better evaluate the role of new direct oral anticoagulants in patients with PNH.

## Abbreviations

GPI: Glycosylphosphatidylinositol
PNH: Paroxysmal nocturnal haemoglobinuria
QOL: The quality of life
VKA: Vitamin K antagonist

## References

[1] Rotoli B, Luzzatto L (1989). Paroxysmal nocturnal hemoglobinuria. Semin Hematol.

[2] Socie G, Gramont A, Rio B, Leporrier M, Rose C, Heudier P (1996). Paroxysmal nocturnal haemoglobinuria: long term follow-up and prognostic factors. Lancet.

[3] Hall C, Richards S, Hilmenn P (2003). Primary prophylaxis with warfarin prevents thrombosis in paroxysmal nocturnal hemoglobinuria (PNH). Blood.

[4] Hillmen P, Muus P, Duhrsen U, Risitano AM, Schulbert J, Luzzatto L (2007). Effect of the complement inhibitor eculizumab on thromboembolism in patients with paroxysmal nocturnal hemoglobinuria. Blood.

[5] van Bijnen ST, Østerud B, Barteling W, Verbeek-Knobbe K, Willemsen M, van Heerde WL (2015). Alterations in markers of coagulation and fibrinolysis in patients with paroxysmal nocturnal hemoglobinuria before and during treatment with eculizumab. Thromb Res.

[6] WHOQOL-BREF - The World Health Organization Quality of Life Official Manual. www.who.int/mental_health/media/en/76.pdf

[7] Eriksson BI, Borris LC, Friedman RJ, Haas S, Huisman MV, Kakkar AK (2008). Rivaroxaban versus enoxaparin for thromboprophylaxis after hip arthroplasty. N Engl J Med.

[8] Patel MR, Mahaffey KW, Garg J, Pan G, Singer DE, Hacke W (2011). Rivaroxaban versus warfarin in nonvalvular atrial fibrillation. N Engl J Med.

[9] Weitz JI, Lensing AWA, Prins MH, Bauersachs R, Beyer-Westendorf J, Bounameaux H (2017). Rivaroxaban or Aspirin for Extended Treatment of Venous Thromboembolism. N Engl J Med.

